# Examining the Mechanisms behind Exercise’s Multifaceted Impacts on Body Composition, Cognition, and the Gut Microbiome in Cancer Survivors: Exploring the Links to Oxidative Stress and Inflammation

**DOI:** 10.3390/antiox12071423

**Published:** 2023-07-14

**Authors:** Benjamin Matei, Kerri M. Winters-Stone, Jacob Raber

**Affiliations:** 1 Department of Behavioral Neuroscience, L470, Oregon Health & Science University, 3181 SW Sam Jackson Park Road, Portland, OR 97239, USA; benjamin.matei@outlook.de; 2 Division of Oncological Sciences, School of Medicine, Oregon Health and Science University, Portland, OR 97239, USA; wintersk@ohsu.edu; 3 Knight Cancer Institute, Oregon Health and Science University, Portland, OR 97239, USA; 4 College of Pharmacy, Oregon State University, Corvallis, OR 97331, USA; 5 Departments of Neurology and Radiation Medicine, Division of Neuroscience ONPRC, Oregon Health & Science University, Portland, OR 97239, USA

**Keywords:** cancer survivor, exercise, quality of life, inflammation, oxidative stress, cachexia, sarcopenia, cognitive impairment, fatigue, gut microbiome

## Abstract

This review focuses on the effects of exercise on various health-related outcomes in cancer survivors, encompassing body composition, cognitive function (including sleep), and gut microbiome health. By analyzing multiple studies, we aimed to summarize the existing evidence and shed light on underlying mechanisms. The findings strongly suggest that exercise serves as a multifaceted non-pharmacological strategy, playing a significant role in improving the overall health of cancer survivors by effectively reducing inflammation and oxidative stress. Exercise plays a crucial role in preventing muscle wasting, diminishing the presence of reactive oxygen species and pro-inflammatory cytokines, and enhancing antioxidant systems. Furthermore, exercise displays notable benefits in terms of executive cognitive functioning and fatigue alleviation, largely attributed to its anti-inflammatory impact on the central nervous system and its ability to induce neurogenesis via growth factors. Additionally, exercise positively influences microbial diversity, reduces gut inflammation, and enhances neurogenesis through the gut–brain axis. Our key findings underscore the reduction of oxidative stress and inflammation as primary mechanisms by which exercise effectively enhances health outcomes in cancer survivors. By delving deeper into these candidate mechanisms, we aim to provide valuable guidance for future research and interventions targeting the symptoms experienced by cancer survivors.

## 1. Introduction

Chronic diseases, such as heart disease, stroke, and especially cancer, are major causes of morbidity and mortality worldwide, particularly with an increasingly aging population [1]. The number of new cancer cases has increased by 33% since 2007, resulting in 24.5 million new cases in 2017 [2]. Cancer treatments have advanced significantly, turning many forms of cancer into chronic conditions and prolonging the lives of cancer survivors many years after their initial diagnosis. In the UK, cancer survival rates have doubled over the last four decades, with 50% of patients surviving for ten or more years [3]. As of 2020, the global 5-year prevalence of cancer was 50.5 million cases [4].

However, the increasing number of cancer survivors frequently suffer from ongoing symptoms, including but not limited to neuropathy, pain, and sleep disturbance [5]. Cancer treatments have been repeatedly shown to disrupt the gut microbiome, leading to various gastrointestinal disorders such as mucositis and colitis, and an increased risk of colon cancer [6,7,8]. Several anticancer agents, known to cause reversible and/or irreversible cardiomyopathies, blood pressure alterations, arrhythmias, vasculitis, and other types of cardiovascular disease (CVD), represent dose-limiting factors for many anticancer treatments [9]. More acute side effects during treatment include nausea, vomiting, and diarrhea [10]. Moreover, cachexia and sarcopenia are frequently observed symptoms among cancer survivors, with significant implications for their outcomes [11,12]. Impaired cognitive functioning, including difficulty concentrating, memory impairment, and increased anxiety, is a common experience even years after treatment for many survivors [13,14,15]. In addition, cancer-related fatigue (CRF) can worsen these impairments, further diminishing the quality of life (QOL) in individuals affected by cancer [16]. Neoplasms moved up the ranks among the top causes of disability-adjusted life-years from sixth place in 1990 to the second-leading cause in 2017 [2].

Oxidative stress, characterized by an imbalance caused by either increased generation of reactive oxygen species (ROS) or a decline in antioxidant defense mechanisms has emerged as a key factor connecting the just-described symptoms in cancer survivors [17,18,19,20,21]. During normal cellular metabolism, ROS are generated as byproducts, predominantly through the mitochondrial respiratory chain [22]. In controlled and moderate levels, ROS participate in cellular signaling pathways, regulating processes such as cell growth, differentiation, and survival, and are important contributors to antimicrobial immunity [23,24].

Chronic and prolonged increases in ROS levels, observed in oxidative stress, however, can result in cellular damage, DNA mutations, and neoplastic transformation, playing a significant role in the initiation and progression of cancer [25,26]. Elevated ROS levels also stimulate the production of pro-inflammatory cytokines such as tumor necrosis factor-α (TNF-α), interleukin-1 (IL-1), and IL-6, and activate the nuclear factor-κB (NF-κB) pathway [26,27]. Excessive oxidative stress, considered a hallmark of cancer, therefore, is associated with several pathological conditions, including chronic inflammation and various types of cancer [17,25,28,29].

Indeed, a robust and well-established connection exists between oxidative stress, chronic inflammation, and the development of cancer [26,27]. Oxidative stress-induced inflammation not only contributes to the development of cancer but also plays a significant role in the manifestation of various debilitating symptoms experienced by cancer survivors, including those we have described previously, as we will explore in the following sections [25].

Given the multitude of short- and long-term consequences of cancer and its treatment, often linked to inflammation and oxidative stress, addressing these factors is crucial for preventing further cancer development, improving the well-being of cancer survivors, and mitigating the occurrence of various pathologic conditions and impairments. Non-pharmacological interventions have great potential as they can serve as a better alternative to drugs, which are expensive and can cause numerous side effects, exacerbating the already challenging symptom burden faced by cancer survivors. Exercise has long been identified as an effective non-pharmacological intervention to improve health outcomes in chronic conditions such as obesity, type 2 diabetes, neurodegenerative diseases, cardiovascular disease, and also in cancer [30,31]. Epidemiological studies have shown that exercise is associated with improvements in treatment-related outcomes and overall survival in cancer patients, while also offering multiple benefits for QOL in cancer survivors [18,31,32,33,34,35,36,37,38,39]. Regular exercise promotes the activation of antioxidant defenses, mitigating the harmful effects of oxidative stress and allowing for the elimination or reduction of oxidative stress before it can cause damage to cellular structures [40]. However, understanding this underlying candidate mechanism and the other mechanisms through which exercise benefits cancer survivors is important in order to confirm the efficacy of exercise-related benefits and to further inform targeted interventions to optimize outcomes.

In this comprehensive review, we will examine the existing evidence regarding the effects of exercise on cancer survivors. We will start with a brief definition of cancer survival and explore the types of exercise included in this review. To differentiate our review from other publications, we will focus on the positive impacts of exercise in three key domains: body composition, cognitive function (including sleep behavior), and gut microbiome. We will attempt to unravel the potential mechanisms that contribute to these benefits, including the intricate interactions involving inflammation and oxidative stress, muscle hypertrophy, neurogenesis, the influence of neurovisceral interactions, and the observed alterations in the gut microbiome [40,41,42,43,44,45,46,47,48,49]. We will also highlight gaps in our current knowledge and provide recommendations for future research in this area. The primary goal of this review is to enhance our understanding of the mechanisms that contribute to the beneficial effects of exercise on cancer survivors. Specifically, it aims to explore the impact of exercise on body composition, gut microbiome, and cognitive outcomes. Furthermore, it aims to emphasize the role of exercise in reducing inflammation and oxidative stress as a shared factor contributing to these effects. This review will further support the role of exercise as an effective non-pharmacological strategy to promote QOL in cancer patients.

## 2. Results

### 2.1. Definition of Cancer Survival and Exercise

A cancer survivor is defined by the National Coalition for Cancer Survivorship as someone who has been diagnosed with cancer and continues to live after their diagnosis [50]. A frequently used synonym for “cancer survivor” in this review is “cancer patient”, primarily indicating individuals who are still undergoing treatment. It is important to note that this distinction may not always be possible or applicable in every context.

The present review investigates various types of exercise and their effects on the health of cancer survivors. For instance, aerobic exercise, including activities such as jogging, cycling, and swimming, is known to improve insulin sensitivity and cardiovascular health [51]. In colon cancer survivors, aerobic exercise enhanced health-related QOL by improving sleep quality, reducing fatigue, and increasing physical functioning and vitality [32]. Resistance exercise, such as weightlifting, on the other hand, improves muscle mass, bone density, and also insulin sensitivity [51]. Multiple studies reviewed in a meta-analysis in 2020 involving breast cancer survivors reported resistance exercise to cause a significant increase in QOL, self-perception, balance, fatigue, and pain [33].

Combining the two types of exercise in certain instances yields even more benefits in certain chronic diseases [35]. Resistance exercise in some cases enhanced the effects that aerobic exercise has on glycemic control and respiratory function in adults [52,53]. Rowing, a cyclic and symmetrical sport combining overall strength with aerobic endurance, was found to significantly improve the physical, emotional, and mental health status of breast cancer survivors, leading to reductions in body fat, fewer limitations in daily routines, enhanced physical activity, improved social life, increased vitality, and overall improvements in QOL parameters, even after a shorter duration of intervention [54]. An increasing number of studies employ the combination of aerobic and resistance exercise to maximize the magnitude and array of benefits, making it challenging to determine specific benefits for each type of exercise.

High-intensity interval training (HIIT), which is mostly anaerobic exercise, is a time-efficient modality consisting of repeated short bursts of intense activity, performed with a “near maximal” or “all-out” effort, followed by periods of rest or low-intensity exercise. HIIT elicits significant physiological and metabolic adaptations, including improvements in insulin sensitivity, cardiovascular health, and body composition [55]. In breast cancer patients, HIIT improved several health outcomes including a reduction in CRF, an increase in health-related QOL, an improvement in muscle strength, and tumor regression after neoadjuvant chemoradiotherapy, as shown in a recent meta-analysis [34]. However, it should be mentioned that there is no universally agreed upon definition for HIIT, so there may be variations in the intensity, duration, and rest periods used in different studies, which could affect the outcomes.

While mind–body exercises such as Pilates, yoga, qigong, and tai chi, have been found to offer certain health benefits to cancer survivors, they pose challenges in standardization and quantifying dosing [36,37,56,57]. These exercises have shown improvements in physical fitness, fatigue, sleep quality, depression, anxiety, and BMI; however, they have not been extensively studied compared to more conventional forms of exercise interventions and often suffer from methodological limitations and a high risk of bias [37]. Before making definite conclusions and recommendations specific to cancer type and symptoms, larger trials with appropriate comparison groups and longer follow-up periods are required with these alternative methods.

Our review adopts a broader, more comprehensive approach to defining exercise and also includes the non-traditional modalities mentioned and physical activity, a range of human movements beyond structured or planned exercise. We aim to distinguish between the effects of different types of exercise and to differentiate between exercise and physical activity whenever possible. However, in some cases, it may not be feasible to make such distinctions and we may rely on our broader category of exercise.

### 2.2. Exercise and Its Effect on Body Composition in Cancer Survivors

Cancer-related wasting syndromes are common and devastating complications in cancer survivors that can have a profoundly negative impact on patients’ survival, QOL, and even their response to treatment [12,58,59]. Muscle wasting syndromes commonly result in poor treatment outcomes, including reduced tolerance to chemotherapy and radiation therapy [60]. The loss of skeletal muscle mass and function frequently leads to reduced physical functioning, overall weakness, and early muscle fatigue, which may be perceived as a feeling of tiredness [61].

Cachexia and sarcopenia are muscle wasting syndromes characterized by an imbalance in protein synthesis and breakdown process [18]. Cachexia is a multifaceted syndrome of muscle wasting, characterized by weight loss primarily from the depletion of skeletal muscle mass, sometimes also accompanied by fat mass loss, that cannot be fully restored through conventional nutritional support [11,58]. According to the up-to-date guidelines, the diagnosis of cachexia requires the presence of at least three additional criteria out of the following contributing factors: fatigue, abnormal biochemistry (including high levels of C-reactive protein (CRP)), decrease in muscle strength, anorexia, and low fat-free mass [62]. Sarcopenia is a distinct muscle wasting syndrome characterized by the loss of muscle mass, decreased strength, and impaired function. It typically affects the elderly population and may not necessarily be accompanied by significant weight loss [63]. Cancer has been recognized in multiple recent studies as the primary contributor to secondary sarcopenia among patients [12].

Both conditions, cachexia and sarcopenia, exhibit inflammation and oxidative stress, although opinions vary, with some sources attributing these characteristics more prominently to cachexia [18,64]. Furthermore, in the context of inflammation, specific regulatory molecules associated with muscle wasting, including members of the ubiquitin–proteasome system (UPS) and myostatin, are activated, while molecules such as insulin-like growth factor 1 (IGF-1) and peroxisome proliferator-activated receptor-gamma coactivator 1 α (PGC-1α) are repressed [18]. A 50% increase in forkhead box protein O1 (FoxO1), a key regulator of the ubiquitin–proteasome system involved in protein breakdown, was observed in tumor-bearing mice compared to control mice with no tumor [65]. Atrogin1, an E3 ligase associated with skeletal muscle wasting, was also observed to be threefold higher in tumor-bearing mice [65]. Significant contributors to muscle wasting include tumor necrosis factor α (TNF-α), prostaglandins, and glucocorticoids (GCs) [66].

It is estimated that approximately half of all cancer patients develop cachexia, with some sources suggesting that up to 80% of those with advanced cancer may suffer from this syndrome [11,67]. Among these patients, a subsequent 1-year mortality rate ranging from 20% to 60% has been observed, highlighting the severity of this symptom in cancer survivors [11]. Notably, a significant proportion of cancer patients, approximately 38.6%, present with pre-therapeutic sarcopenia, with esophageal and small-cell lung cancers demonstrating the highest occurrence [12]. In locally advanced esophageal cancer, the prevalence ranges from 16% to 35% and is linked to unfavorable surgical outcomes and decreased survival. Sarcopenia prevalence reaches 52.8% in lung cancer and is associated with lower chemotherapy response and poorer progression-free survival [68].

A recent study published in the Radiotherapy and Oncology Journal demonstrated the significant advantages of progressive resistance training on lean body mass in individuals with head and neck cancer [69]. Following radiotherapy, a 12-week exercise program was implemented, comprised of 2–3 sets of 8–15 repetitions at maximum capacity for seven conventional exercises. The results indicated a notable increase in lean body mass, along with improved muscle strength and QOL [69]. Additionally, a review focusing on prostate cancer patients undergoing androgen deprivation therapy found that structured and progressive resistance exercise programs effectively attenuated the loss of lean muscle mass commonly observed in these patients [70].

Thus, exercise emerges as a promising intervention to prevent muscle wasting in the context of sarcopenia and cachexia among cancer patients. However, it is important to note that these studies were primarily carried out on non-cachectic cancer patients, or at least without a mention of the patients’ current body composition. Additionally, there is a scarcity of robust studies specifically examining exercise in cancer patients suffering from muscle wasting, making it challenging to draw definitive conclusions regarding the impact of exercise on relieving cancer cachexia or sarcopenia. Further research is warranted to draw definitive conclusions regarding the effects of exercise on muscle wasting in cachectic or sarcopenic cancer survivors. This area of study presents intriguing possibilities for gaining deeper insights into the potential benefits of exercise in these conditions. By examining the mechanisms by which exercise improves body composition in both cancer survivors and healthy individuals, our goal is to optimize exercise interventions and enhance exercise’s effects, especially for survivors suffering from cachexia or sarcopenia.

The most apparent way by which exercise prevents muscle wasting and preserves lean mass in cancer survivors is through exercise-induced muscle hypertrophy, where protein synthesis rates exceed protein degradation rates [71]. Muscle hypertrophy involves the enlargement of muscle fibers, mainly driven by increased protein synthesis rates. This process is associated with the activation of phosphoinositide-3 kinase (PI3K) and the mammalian target of rapamycin (mTOR) [41]. mTOR serves as the primary signaling pathway through which exercise training stimulates protein synthesis and, therefore, muscle hypertrophy. Mechanical stimuli, including resistance training, can activate mTOR complex 1 (mTORC1) signaling. The upregulation of mTORC1 in turn, leads to the phosphorylation of key proteins involved in muscle protein synthesis and hypertrophy, such as p70S6K1 and 4E-BP1 [72]. Similarly, exercise-induced upregulation of mTORC2, along with the direct effect of mTORC1 activation on protein synthesis, may contribute to the attenuation of muscle atrophy. The activation of mTORC2 stimulates protein kinase B, also called Akt, leading to the downregulation of the UPS, thereby further protecting against muscle loss [73]. Resistance training in healthy individuals reduced muscle FoxO1 and increased Akt and mTOR phosphorylation, while detraining had the opposite effect, decreasing Akt phosphorylation and increasing FoxO1 levels [74]. A single bout of resistance exercise has been shown to decrease Atrogin-1 mRNA after 48 h, thereby already showing potential to prevent muscle wasting [75,76]. It should be noted that this was observed in a healthy population and may not be directly applicable for cancer survivors. Furthermore, in the context of cancer, additional contributing factors such as alterations in metabolism or heightened inflammation and oxidative stress may potentially diminish the magnitude of the observed effects of muscle hypertrophy.

Exercise has also shown to be an effective strategy for preventing muscle atrophy by reducing both systemic and local (skeletal muscle) inflammation [77]. This may be of particular importance, as systemic inflammation has been found to enhance protein degradation via the UPS and decrease protein synthesis in skeletal muscle [18,78]. One of the numerous highly reactive molecules and free radicals that serve as mediators of inflammation are ROS, as we already discussed. ROS also participate in muscle catabolism via the UPS and were shown to increase the production of 4-hydroxynonenal (HNE) [18]. HNE is not only a simple marker of oxidation but also a direct cause of oxidative stress. Overaccumulation of ROS and HNE lead to oxidative stress and systemic inflammation, eventually contributing to an increase in cancer risk and to muscle atrophy, as already mentioned [78,79]. Similarly, TNF-α also plays a crucial role in initiating the inflammatory response [80]. Lower levels of TNF-α and other pro-inflammatory cytokines, such as IL-6, were observed in university students who participated in moderate-intensity exercise for six weeks during their midterm, compared to the control group. However, another group of students participating in HIIT as part of the same study, showed heightened levels of TNF-α and IL-6 [81]. Since the exercise duration was only six weeks, the study participants may not have acclimated to the strenuous exercise by that point, potentially leading to an exaggerated inflammatory response. A single intensive bout of exercise can cause an overproduction of ROS leading to oxidative stress and potentially tissue damage [82]. On the contrary, regular exercise, as observed in breast cancer patients, allows the body to adapt to stress, enabling it to respond swiftly and prevent or minimize the harmful effects of oxidative stress on cellular structures. Consequently, maintaining consistent exercise habits appear to provide antioxidant protection [83,84]. Similarly, appropriate exercise, particularly moderate to high intensity exercise can induce adaptive responses and enhance the body’s natural antioxidant defense systems. This helps maintain muscle redox balance and combat excessive ROS [42]. Considering that increased inflammation is a hallmark of cancer, we hypothesize that in cancer survivors, the impact of reducing inflammation assumes even greater significance, considering their heightened inflammatory state [28].

Another hypothesis suggests that GCs play a major role in muscle wasting via the hypothalamic–pituitary–adrenal glucocorticoid axis [66]. GCs cause decreased protein synthesis and an increase in protein degradation in skeletal muscle, exactly the misbalance that can be observed in cancer cachexia and sarcopenia [85]. This effect is attributed to a transcriptional program stimulated by GCs. This program decreases the expression of genes promoting protein synthesis and increases the expression of those promoting protein degradation, such as FoxO1 or Atrogin-1 [86,87]. During exercise, the body experiences a natural stress response, which leads to an increase in GC levels, measured primarily through cortisol. This is a physiological response that helps to maintain an euglycemic state. Larger amounts of cortisol are produced during intense and unfamiliar exercise sessions leading to effects similar to the ones described with the overproduction of ROS [88]. Chronically increased levels of GC have been associated with a manifold of symptoms and can even potentially contribute to neurodegeneration and impair brain function [89]. Three studies that investigated exercise interventions in breast cancer patients demonstrated a significant impact on cortisol levels, resulting in a decrease in its concentration [43,90,91]. This may initially appear paradoxical, as the physiological response during stress is to elevate cortisol levels, as we just elaborated, however, this elevation is only temporary. These studies revealed that exercise leads to a progressive reduction in cortisol levels over time. By progressively reducing GCs over time, exercise can counteract the mechanism that causes decreased protein synthesis and increased protein degradation in skeletal muscle [85]. In this case, the same effect described previously applies once again. Regular exercise leads to the body adapting to stress [83]. Moreover, regular exercise can even contribute to reducing the body’s stress levels over time, as supported by the observed progressive reduction of GCs in breast cancer patients who engaged in exercise [43,90,91].

Exercise may also be effective in addressing the psychological effects of cachexia, such as depression and anxiety. By doing so, it can contribute to improved nutritional intake, promote a healthier lifestyle, and provide further benefits to individuals coping with cachexia [92]. 

In conclusion, exercise has shown significant potential in improving lean mass and potentially preventing muscle wasting in cancer survivors. This is accomplished through various mechanisms, including increased antioxidant defenses, reduced inflammation and cortisol levels over time, as well as the promotion of muscle hypertrophy and potentially psychological influence. It is important to note that the effects on the body can vary depending on the specific type of exercise performed. Endurance training promotes oxidative metabolic adaptations with minimal impact on muscle mass. However, if the intensity is very high, it may temporarily upregulate atrogene signaling or increase GC levels [78,81]. Resistance training exerts an anabolic effect, leading to muscle hypertrophy and may decrease signaling for ubiquitin proteasomes [78,93]. Combining resistance and aerobic muscle training has been proposed to be included as part of cachexia treatment programs [94]. The combination shows promise, but the intensity should be adjusted based on the individual’s fitness level to avoid excessive buildup of ROS or cortisol, to find the right balance that can potentially lead to an overall reduction of GCs and a sufficient increase in antioxidants.

### 2.3. Exercise and Its Effect on Cognitive Outcomes in Cancer Survivors

#### 2.3.1. Cancer-Related Fatigue

Experienced by cancer survivors throughout all stages of the disease trajectory, fatigue emerges as a particularly severe symptom with profound and wide-ranging effects on their physical, emotional, and social well-being, significantly impairing their overall QOL [19,95]. It also has a significant impact on the ability to tolerate various treatment modalities, often leading to the discontinuation or delay of treatment [96].

Fatigue is commonly described as an overwhelming sensation of tiredness, lack of energy, and a persistent feeling of exhaustion, resulting in difficulties in carrying out certain tasks [95]. Fatigue encompasses both mental and physical aspects, with mental fatigue relating to cognitive or perceptual aspects, and physical fatigue relating to the performance of the motor system. While mental and muscular fatigue are distinct conditions, they can share physical effects, making it challenging to differentiate between the two [19]. Fatigue is also an integral component of the symptom complex associated with cachexia, as it is encompassed within the broader definition of cachexia that requires meeting a minimum of three out of five additional criteria for diagnosis, as we already elaborated. Another of these criteria involves abnormal biochemistry, such as elevated CRP [62]. According to reviews, increased neutrophil and monocyte counts, along with elevated levels of various inflammatory markers such as IL-6, IL-1β, and CRP, have been found to be correlated with fatigue in cancer survivors [97].

The reduction in BMI which is observed in cachexia specifically, is known to additionally contribute to severe fatigue in cancer survivors [67]. As already mentioned, the loss of skeletal muscle mass and the resulting weakness is often perceived as fatigue or tiredness [61]. We hypothesize that inflammation and oxidative stress play a significant role in the development of CRF. This hypothesis is supported by several other studies investigating the pathogenesis of CRF [19,95]. A similar hypothesis has been suggested for the association between inflammation and depression together with fatigue [98]. This connection aligns with the observation that depressed patients often experience fatigue and a lack of drive as prominent symptoms [99].

CRF is frequently observed among survivors, with reported rates ranging from 62% to 85% in individuals undergoing active treatment. Among those affected, moderate to severe levels of CRF are reported by 9% to 45% [100,101]. Moreover, long-term adult survivors of various cancers, such as cervical, lower gastrointestinal, breast, lymphoma, and mixed cancers, also experience chronic CRF with rates ranging between 23% and 49% [95].

A systematic review of 15 studies found that certain mind–body exercises have the potential to improve outcomes related to fatigue, sleep quality, depression, and anxiety in cancer survivors [37]. The review demonstrated a significant overall relief of fatigue with mind–body exercise, and half of the reviewed studies showed a significant improvement in sleep quality [37]. In a randomized controlled trial, medical qigong intervention demonstrated a clinically significant reduction in CRF in the intervention group [36]. Similarly, a study with high-risk prostate cancer patients participating in physical exercise demonstrated improvements in fatigue along with a decrease in pro-inflammatory cytokines such as IL-1, IL-6, and TNF-α [38]. Another study involving older females receiving hormonal treatment for breast cancer found that a walking exercise intervention tended to improve sleep quality, as indicated by decreased wake time at night and reduced movement during sleep [43]. Additionally, the study conducted with colon cancer patients that was previously mentioned to illustrate the benefits of aerobic exercise, reported notable improvements in fatigue and sleep quality [32].

Mind–body exercise and more conventional forms of exercise offer multiple mechanisms to alleviate CRF and pathological sleep behavior. One of the main mechanisms is the already described anti-inflammatory effect and reduction in oxidative stress that follows exercise. The study investigating the effects of medical qigong on CRF found a significant decrease in CRP after a ten-week intervention comprising two supervised 90-min sessions per week [36]. The observed decrease in pro-inflammatory cytokines in prostate cancer patients after an exercise intervention further reinforces these findings [38]. CRP is an acute-phase protein known to be a marker of inflammation, and as previously established, its elevation is considered part of the symptom complex of cachexia [62]. The production of CRP is influenced by the pro-inflammatory cytokines IL-1, IL-6, and TNF-α [102]. Given the established association between these cytokines and CRP with fatigue, it is reasonable to expect that reducing these inflammatory markers would lead to an improvement in fatigue [97]. Regular exercise in breast cancer survivors after primary treatment has consistently shown to decrease pro-inflammatory cytokines (IL-2, IL-6, IL-8, TNF-α) and increase anti-inflammatory cytokines (IL-10) [103]. By targeting inflammation and related prognostic biomarkers of cancer, including CRP and TNF-α, exercise holds promise for reducing and preventing CRF.

Mind–body exercise demonstrates a positive impact on the emotion-related network, affecting specific brain systems involved in these functions [104,105]. The amygdala, involved in processing emotionally significant stimuli and facilitating information exchange between the prefrontal-temporal association cortices and the hypothalamus, demonstrates decreased activation in response to mind–body exercise [106]. Additionally, mind–body exercises have the potential to inhibit stress responses and modulate affective, autonomic, hormonal, and immune processes through neurovisceral feedback, thereby enhancing cell-mediated and mucosal immunity [44,107]. These findings underscore the potential of mind–body exercise in addressing psychological challenges and managing stress associated with a cancer diagnosis. This, in turn, promotes improved social and emotional well-being among cancer survivors and may ultimately contribute to better sleep [108].

By targeting inflammation through exercise, not only can CRF potentially be mitigated, but the benefits may extend to addressing fatigue and depression in a broader context. Addressing depression through exercise’s anti-inflammatory properties would further contribute to promoting emotional well-being among cancer survivors. The observed mechanisms discussed in exercise’s effects on body composition might also come into play as fatigue is closely linked to cachexia and sarcopenia. Incorporating exercise or physical activity into comprehensive care strategies offers a promising approach to managing CRF and promoting overall well-being in cancer survivors.

#### 2.3.2. Cancer-Related Cognitive Impairment

Cancer-related cognitive impairment (CRCI) is a recognized side effect of cancer and its treatment, affecting patients of all ages [13]. These cognitive impairments can have a significant impact on treatment decisions and on the QOL of cancer survivors, persisting even years after treatment [14,15].

CRCI encompasses deficits in various cognitive domains, including learning, memory, attention, processing speed, and executive function. Executive function, controlled by the prefrontal cortex, is responsible for regulating cognitive processes such as planning, organizing, and verbal fluency [15]. It should be noted that CRCI can present in various ways, impacting different cognitive domains. Its effects can range from subtle to significant, with durations that may be temporary or permanent, and a progression that can be stable or progressive [109].

Sleep duration and quality are considered important factors that can significantly influence cognitive function [16]. A recent meta-analysis revealed that variations in sleep duration and quality are among the strongest predictors of cognitive decline. This suggests that the quality and duration of sleep have a substantial impact on cognitive abilities, highlighting the importance of maintaining optimal sleep patterns for optimal cognitive function [16]. Chronic inflammation and neuroinflammatory pathways are believed to play a significant role in cognitive impairment, particularly in the context of cancer [15]. Cancer treatment can induce injury to the central nervous system (CNS) by damaging blood vessels, glial cells, and through the processes of neuroinflammation and oxidative stress [20]. This is also seen by elevated levels of inflammatory components in CRCI, including an increase in pro-inflammatory cytokines such as IL-1, IL-6, and TNF-α [15]. Radiotherapy may additionally impair the neurogenesis of cells in the hippocampus [110].

During treatment, up to 75% of patients experience CRCI, and even after completing cancer treatments, up to 35% of patients may continue to experience CRCI for months or even years [109,111]. Our understanding of the molecular mechanisms behind cognitive difficulties remains limited. However, by gaining a deeper understanding of how exercise benefits survivors experiencing these symptoms, we can enhance our understanding of the underlying causes and develop strategies to improve or prevent cognitive impairment in cancer survivors.

Healthy older adults undergoing a 12-week program consisting of two 30-min cycling sessions per week at 60% of their theoretical maximal heart rate showed improvements in executive-level cognitive functions such as flexibility and working memory [45]. These observed benefits, although observed in healthy individuals, are precisely the advantages that could be beneficial for cancer survivors suffering from CRCI. A systematic review was conducted to examine the effects of physical and mind–body exercise on cognitive function in cancer patients. Out of the trials analyzed, 13 trials (45%) reported a significant benefit of exercise in improving cognitive function compared to control groups [112]. Specifically, self-reported cognitive function demonstrated significant enhancement in the majority of these trials. Moreover, some trials showed favorable effects of aerobic exercise or combined aerobic and resistance exercise on objective neuropsychological tests assessing cognitive function [112]. Although further research is necessary in cancer survivors specifically, these findings suggest that both physical activity and mind–body exercise may have a positive impact on cognitive impairments associated with cancer and its treatment.

Research has demonstrated that exercise can enhance antioxidation and counteract the adverse effects of oxidative stress in cancer survivors by regulating systemic oxidative status and DNA repair capability [113]. As a result, engaging in regular exercise increases the body’s ability to withstand oxidative challenges and mitigate oxidative stress and the often-resulting inflammation. Given the significant involvement of chronic inflammation and neuroinflammatory pathways in CRCI and the observed elevated levels of pro-inflammatory cytokines such as IL-1, IL-6, and TNF-α, exercise has the potential to offer significant benefits in mitigating chemotherapy-induced cognitive impairment [15,20]. Exercise may primarily benefit survivors suffering from CRCI by exerting overall anti-inflammatory effects on the CNS, thereby moderating therapy-induced inflammation. However, the beneficial effects of exercise on survivors with CRCI extend beyond its anti-inflammatory effects alone. Other mechanisms also contribute to its positive impact.

As previously explored in detail, GC levels are closely intertwined with exercise [66,114,115]. Moreover, studies have provided evidence that adrenalectomy and basal-level corticosterone replacement in aged rodents attenuated hippocampal pathology, indicating the involvement of elevated GC levels in hippocampal atrophy [116]. Regular exercise has been shown to reduce GC levels in cancer survivors over time, suggesting its potential to counteract hippocampal atrophy and promote hippocampal functioning [117]. This notion is supported by a research article published in the Hippocampus journal, which has reported that exercise enhances hippocampal-dependent cognitive function in rats, accompanied by increased levels of brain-derived neurotrophic factor (BDNF) protein in the dentate gyrus, hippocampus, and perirhinal cortex [49]. However, further research is needed to validate these findings in human studies and fully understand their implications. This investigation may shed light on the connection between exercise, the HPA axis, and the consequences for GC levels.

In a study conducted with cancer patients currently undergoing treatment, it was also found that exercise led to an increase in BDNF levels [46]. BDNF, therefore, may provide another plausible explanation for the observed cognitive benefits in exercise-related studies. However, it is worth noting that several other molecules, such as vascular endothelial growth factor (VEGF) or IGF are also released during exercise [45]. These molecules, along with BDNF, are considered growth factors and collectively contribute to processes such as angiogenesis, synaptogenesis, and neurogenesis, thereby promoting brain plasticity and enhancing neurovascular integrity [118]. These growth factors have the potential to induce structural changes in the cerebral vasculature, leading to increased volumes of the gray nuclei, hippocampus, and gray and white matter [45].

Regular exercise has been demonstrated to serve as a preventive measure against treatment-related CNS injuries in cancer survivors [20,110]. Exercise has been shown to enhance antioxidant defenses, decrease GC levels, enhance hippocampal-dependent cognitive function, and promote the release of growth factors such as BDNF, VEGF, and IGF, which contribute to neurovascular integrity and induce neurogenesis [43,45,117,118]. In conclusion, exercise appears to play a significant role in reducing CRCI and CRF, whether related to treatment or in general. Finally, we will explore how exercise can induce changes in the gut microbiome, contributing to enhanced neurogenesis, and investigate the underlying mechanisms involved in this process.

### 2.4. Exercise and Its Effects on Gut Microbiome in Cancer Survivors

In cancer patients undergoing chemotherapy, negative changes in brain functioning, cognition, and fatigue, along with the presence of gastrointestinal symptoms, can also be attributed to disruptions in the gut microbiome [119,120]. Both radiation therapy and chemotherapy have been shown to disrupt the gut microbiome, leading to dysbiosis, inflammation, and a weakened immune response [6,121]. These effects can have long-lasting consequences, significantly impacting the QOL of cancer survivors and their motivation to adhere to treatment [119]. A low microbial diversity in the gut has been linked to several chronic diseases, including cancer [7,8,122]. In contrast, increased microbial diversity in the gut has been associated with healthy aging and improved overall health in older individuals [123].

The gut microbiome is a complex community of microorganisms that plays a crucial role in various physiological processes and overall health. It engages in fermentation, breaking down partially digested complex carbohydrates and yielding short chain fatty acids (SFCAs) such as butyrate, acetate, and propionate [124]. Among the SCFAs, butyrate stands out for its critical role in maintaining gut homeostasis and supporting gastrointestinal well-being [125]. Butyrate possesses multifaceted properties that contribute to cellular homeostasis, including anti-inflammatory, antioxidant, and anticarcinogenic effects [126]. Furthermore, it exerts trophic effects on the colon mucosa by promoting intestinal mucin production and influencing the expression of tight-junction proteins. These combined actions result in the enhancement of intestinal barrier functions, potentially leading to a positive impact on intestinal permeability [127]. SCFAs, including butyrate, exhibit the ability to directly interact with the hypothalamus by crossing the blood–brain barrier (BBB), providing energy for brain microglia, and exerting neuroprotective effects [128]. These interactions occur through the bi-directional mechanisms of the gut–brain axis, which involve vagal nerve innervation, neurotransmitter production, endocrine signaling, and inflammation [129,130]. Key molecules involved in this bidirectional exchange, such as SCFAs and tryptophan, engage with enteroendocrine cells, triggering activation of the vagus nerve—one of the 12 cranial nerves [131]. The gut microbiome, through its involvement in nutrient and mineral absorption, enzyme synthesis, amino acid and neurotransmitter production, as well as the generation of metabolites such as SCFAs, plays a significant role in influencing brain functioning, cognition, and behavior in both rodents and humans [132,133].

Low microbial diversity has been identified as a significant factor in contributing to carcinogenesis [8]. However, the precise mechanisms by which bacteria contribute to this process are not fully understood. It is believed that chronic inflammation, immune dysfunction, elevated levels of lipopolysaccharides, variations in bile acid composition, and increased intestinal permeability (leaky gut) are among the mechanisms involved [134]. Observational studies conducted on patients with breast cancer undergoing chemotherapy treatment reveal a correlation between inflammation and the emergence of behavioral symptoms [120]. Additionally, there is evidence that gut microbes could potentially contribute to the development of both peripheral and central inflammation as a response to chemotherapy treatment, ultimately making them contribute to behavioral symptoms and gut inflammation [119].

Nearly 90% of colorectal cancer cases are sporadic, while the remaining cases are attributed to genetic and environmental factors. Lifestyle factors, including physical inactivity, smoking, unhealthy dietary habits, alcohol consumption, and obesity, have been identified as key risk factors for colorectal cancer, and these factors are known to influence the composition of the gut microbiota [8,135] As mentioned earlier, cancer survivors often experience symptoms linked to the gut microbiome, making exercise an important factor to consider for addressing such symptoms. Exercise is a significant modulator that exerts a positive influence on the gut microbiome and holds potential benefits for cancer survivors. Emerging evidence suggests that the influence of exercise on the gut microbiota may be independent of the consumed diet [136].

Moderate exercise has been found to impact the gut microbiome composition and function, resulting in the production of beneficial metabolites such as butyrate [137,138]. Additionally, acute exercise has been found to enhance the activity of a specific monocarboxylate transporter, facilitating the passage of butyrate across the BBB [128]. In animal studies, the administration of sodium butyrate following cerebral ischemia has been shown to increase the number of cells expressing specific neural proteins, including polysialic acid-neural cell adhesion molecule, nestin, glial fibrillary acidic protein, and BDNF, in various brain regions. As a result, there was a notable enhancement in neurogenesis within the ischemic brains of the mice [139]. Despite this knowledge, there have been few human studies on the effects of exercise on the gut microbiome in cancer survivors, specifically.

One such study was conducted in 2022 and used the Alberta Cancer Exercise (ACE) program to examine its potential in modifying the gut microbiota of 10 breast cancer survivors who had undergone chemotherapy [140]. The study investigated the impact of post-exercise gut microbiota, either alone or in combination with prebiotic fiber supplementation, on breast cancer outcomes using fecal microbiota transplant in germ-free mice. Although minimal alterations in gut microbial composition were observed in the cancer survivors following ACE, the mice colonized with post-exercise microbiota exhibited a consistent trend of reduced tumor volume over time compared to those colonized with pre-exercise microbiota, with significant differences observed on days 16 and 22 [140]. Although the sample size was limited, certain participants demonstrated an augmentation in alpha diversity following exercise. Certain individuals, therefore, may exhibit a more pronounced gut microbial response to exercise compared to others [140].

Another study conducted in 2022 on a larger sample size of patients with colorectal cancer did not involve an exercise intervention [141]. Instead, it assessed the participants’ physical activity levels through a questionnaire and analyzed their microbial profile. The study revealed a positive correlation between higher levels of physical activity and greater diversity of the gut microbiome. Interestingly, there were variations in the results based on sex and tumor site, highlighting the need for further research and detailed analysis [141]. Given that increased microbial diversity is linked to overall host health, these results highlight the potential link between cardiorespiratory fitness and a favorable gut microbiome [125,142]. Exercise was found to enhance the abundance of beneficial bacterial genera, such as Faecalibacterium and Akkermansia, while reducing the levels of potentially harmful genera such as Fusobacterium, ultimately fostering an anti-inflammatory phenotype [143]. Conversely, physical inactivity has been associated with reduced microbial diversity and a less healthy gut microbiome, underscoring the importance of exercise in maintaining optimal gut microbiome health [143].

The precise mechanism through which exercise or physical activity promotes microbial diversity is not yet fully understood, but it is hypothesized that multiple factors contribute to this phenomenon. Active individuals are more likely to have exposure to diverse environmental biospheres and tend to adopt healthier overall lifestyles compared to non-active individuals [142]. These factors may contribute to the presence of a more diverse gut microbiome in physically active individuals. Exercise-induced changes in the gut microbiome have the potential to create an environmental setting that may foster a richer microbiota diversity [144].

The effect of exercise on reducing inflammation in the gut is likely attributed to its beneficial impact on the composition of the gut microbiome and the already described anti-inflammatory phenotype [137,143]. A less diverse microbial profile is associated with a hyperinflammatory state, so it is logical to assume that exercise, by promoting a more diverse gut microbiome, would help reduce inflammation by fostering diversity [127]. These findings are supported by evidence showing that exercise promotes the growth of specific bacterial taxa that have a higher likelihood of producing butyrate, as already established [145]. Butyrate is known for its anti-inflammatory properties and plays a crucial role in suppressing gut inflammation [126]. By increasing the presence of these beneficial bacteria, exercise has the potential to enhance butyrate production, leading to a reduction in inflammation and its associated detrimental effects, such as gastrointestinal symptoms commonly experienced by cancer survivors [39,146].

Furthermore, individuals with higher VO2peak, which represents the maximum rate of oxygen consumption during intense exercise, demonstrated reduced biosynthesis of lipopolysaccharide (LPS), a major component of Gram-negative bacteria cell walls and an endotoxin when present in the blood [142]. Elevated blood LPS levels have been linked to chronic low-level inflammatory states associated with the creation of a favorable immunosuppressive microenvironment for tumor progression [21]. Exercise training has been shown to attenuate inflammation by reducing LPS levels in the blood [147]. Studies in both mice and humans have consistently shown that exercise decreases plasma LPS concentrations, highlighting the beneficial effects of physical activity on reducing LPS biosynthesis and promoting an anti-inflammatory state [147,148]. Exercise has been shown in preclinical studies to enhance the upregulation of critical antioxidant enzymes, including catalase and glutathione peroxidase, providing enhanced protection against oxidative stress and gut inflammation [145]. It also enhances the production of anti-inflammatory cytokines such as IL-10 in intestinal lymphocytes, while decreasing proinflammatory cytokines such as TNF-α [149]. These changes contribute to an overall improvement in immune regulation.

Recent findings indicate that the positive impacts of exercise on systemic metabolic health may be attributed, at least in part, to the influence exerted on the hypothalamus [150]. The influence exercise has on the gut microbiota composition also leads to changes in specific bacterial strains, such as Lactobacillus johnsonii and L. reuteri [151]. L. reuteri is associated with brain health as it is known to produce Vitamin B12, which is needed for the development, myelination, and function of the CNS [127]. Furthermore, exercise, whether performed independently or in conjunction with other interventions such as diet and psychotherapy, has demonstrated the ability to regulate the levels of neuropeptides, including a decrease in neuropeptide Y and an increase in α-MSH, in animal models as well as in overweight and obese individuals [152]. These findings suggest enhancements in hypothalamic function as a result of exercise [153]. However, further research is needed to better understand the underlying mechanisms and to validate these findings through more comprehensive assessments of hypothalamic functionality. Interestingly, butyrate has the ability to enhance the expression of BDNF in the hippocampus and frontal cortex, promoting the survival of neurons, as well as facilitating the generation of new neurons and the formation of synapses [154].

Exercise has been shown to influence the gut microbiome by promoting microbial diversity and the production of beneficial metabolites such as butyrate. Physical activity is associated with a more diverse gut microbiome, which can help reduce inflammation and its detrimental effects [143]. Exercise also has positive effects on hypothalamic function and neuropeptide regulation, potentially impacting brain health [154]. Further research is needed to fully understand the mechanisms underlying these effects and to explore the specific impact of exercise on the gut microbiome in cancer survivors. Nevertheless, these findings highlight the potential of exercise in promoting a healthy gut microbiome and improving overall well-being.

## 3. Discussion

**Figure 1:**
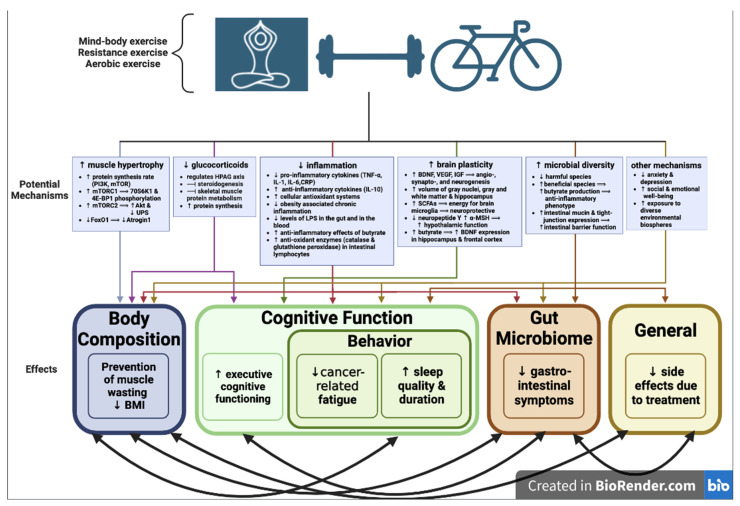
A comprehensive summary illustrating the impact of exercise on cognitive function, behavior, body composition, and the gut microbiome in cancer survivors, demonstrating potential underlying mechanisms. Created with BioRender.com. Abbreviations: Mammalian target of rapamycin (mTOR); phosphoinositide-3 kinase (PI3K); mammalian target of rapamycin complex 1 (mTORC1); mammalian target of rapamycin complex 2 (mTORC2); ubiquitin–proteasome system (UPS); forkhead box protein O1 (FOXO1); hypothalamic–pituitary–adrenal glucocorticoid (HPAG); tumor necrosis factor α (TNF-α); C-reactive protein (CRP); lipopolysaccharide (LPS); brain-derived neurotrophic factor (BDNF); vascular endothelial growth factor (VEGF); insulin-like growth factor (IGF); short-chain fatty acid (SFCA); body mass index (BMI).

In order to present a comprehensive understanding of the intricate relationship between exercise, body composition, cognitive function, sleep behavior, and the gut microbiome in cancer survivors, Figure 1 depicts the key points discussed thus far. This visual representation encompasses various aspects, highlighting the anti-inflammatory properties of exercise and its significant role in benefiting cancer survivors across all of these domains. Additionally, the figure summarizes the potential mechanisms through which exercise can alleviate symptoms in cancer survivors, such as cancer cachexia, sarcopenia fatigue, cognitive decline, and treatment-related gut disruption. It also illustrates the interconnectedness of these beneficial effects. By summarizing these effects and mechanisms, the figure serves as a valuable reference for further discussions, emphasizing the crucial role of exercise in promoting the well-being of cancer survivors.

In the following discussion, we will delve deeper into conflicting study results, the current gaps in knowledge, and propose future directions to advance our understanding of the effects of exercise in the discussed domains and its implications for cancer survivors. By addressing these research gaps, we can pave the way for evidence-based recommendations and interventions that optimize the benefits of exercise on gut health and contribute to improved well-being.

Studies differ in their view whether exercise provokes an increase in circulating cortisol or overall causes a reduction in cortisol levels. A study measuring 24-h cortisol levels after multiple daily moderate- or high-intensity exercises found a significant increase in cortisol levels after these sessions [88]. Interestingly, despite the elevated cortisol levels throughout the day in the moderate- and high-intensity exercise groups, during the night, the cortisol levels were lower compared to the control group [88]. This suggests that exercise can influence the diurnal rhythm of cortisol secretion, leading to lower cortisol levels during nighttime. Another study found the same increase in GCs after moderate- to high-intensity exercise. With low-intensity exercise, on the other hand, they found a decrease in circulating cortisol levels [155]. The three previously mentioned studies in breast cancer patients demonstrated a general reduction in cortisol levels following exercise interventions, which encompassed various activities such as mind–body exercises and moderate aerobic exercise. However, it is important to note that certain studies conducted on breast cancer survivors have reported no significant difference in cortisol levels between the intervention group and the control group [117]. These findings contradict the observations of studies indicating that moderate exercise results in an overall reduction of cortisol levels. These discrepancies may be attributed to variations in cortisol measurement methods or the timing of cortisol level assessments, considering the circadian rhythm of cortisol secretion.

While most of the studies examined in this review demonstrate the positive impact of physical exercise on fatigue and cancer-related QOL, it is worth noting that recent randomized controlled trials have reported contrasting findings, indicating that fatigue and QOL may not always be improved with physical exercise, or at least not significantly [156,157]. These conflicting results highlight the need for further research and exploration to better understand the potential variability in the effects of exercise on these outcomes in cancer patients and survivors. By delving further into the underlying mechanisms, it may be possible to elucidate the reasons for this variability, enabling the adaptation of exercise interventions that effectively alleviate CRF in a majority of patients.

A meta-analysis examining the impact of exercise on cognition revealed limitations in the assessed studies, such as low statistical power, selective inclusion, publication bias, and variations in data analysis decisions [158]. The meta-analysis critically examined 24 meta-analyses, which collectively claimed an overall positive effect of exercise on cognitive function. However, upon closer examination by the overall meta-analysis, it was determined that the exercise-related benefits were only modest. Moreover, the study emphasized that these benefits significantly diminish when considering key factors and correcting for publication bias. Consequently, the meta-analyses underscored the importance of exercising caution when making assertions or recommendations regarding the cognitive advantages of regular physical exercise in the healthy human population [158].

It is important to note, however, that the data presented in this comprehensive study demonstrate significant variability in effect sizes. Some meta-analyses reported effects approaching 0.5, suggesting that there may be studies with even larger effect sizes as these pertain to the means. This variability underscores the complexity of studying the effects of exercise on cognitive outcomes and emphasizes the need for consistent statistical analysis of the results. A randomized clinical trial, which incorporated many of the positive aspects of study design mentioned in the meta-analysis, was able to demonstrate a significant improvement in cognition due to exercise [159]. Another crucial consideration regarding exercise’s potential impact on cognitive health is its cumulative effect. Engaging in exercise or physical activity and reducing physical inactivity has shown evidence of reducing dementia risk [160]. Additionally, regular physical activity and exercise have been shown to influence risk factors for various conditions, including obesity, hypertension, and diabetes, which may contribute to an additional reduction in dementia risk.

According to the hypothesis proposed by Eduard Kraft, physical activity plays a role in promoting synaptic plasticity and neurogenesis by influencing neurotrophic factors such as BDNF or IGF-1. Cognitive stimulation, on the other hand, guides synaptic plasticity in specific brain regions activated during cognitive tasks, selecting the most active synapses. When these two forms of stimulation are combined, they facilitate the incorporation of new neurons and the formation of fresh synapses, resulting in the restructuring of neural networks. In essence, physical stimulation acts as a catalyst for the effects of cognitive stimulation, enabling the reorganization of the neural network [161]. This suggests that exercise, in combination with cognitive stimulation, would be even more efficient as they work together synergistically.

In a groundbreaking study, the direct administration of BDNF into the brain’s ventricles of rats replicated the positive effects on nonspatial learning observed with exercise, highlighting a promising approach to mimic exercise-related benefits using targeted BDNF administration [49]. A separate study successfully replicated the beneficial effects of exercise on mice with Alzheimer’s disease by genetically and pharmacologically inducing adult hippocampal neurogenesis and increasing BDNF levels in mice [162]. By artificially enhancing BDNF levels and stimulating neurogenesis, researchers may unlock new possibilities for therapeutic interventions aimed at improving cognitive function in cancer survivors or patients suffering from neurodegenerative disease.

It is worth noting that there is limited research on the effects of exercise on the gut microbiome in cancer survivors, with only two studies identified by us meeting these criteria [140,141]. Additionally, many of the studies examining the gut–brain axis in relation to exercise have primarily utilized animal models, which warrants caution when extrapolating findings to cancer survivor patients. Interestingly, exercise-induced changes in the gut microbiome of sedentary adults can be reversed after six weeks of physical inactivity, suggesting that these changes may not be permanent [136]. Another crucial aspect to consider when evaluating studies on the gut microbiome is the mode of exercise, as the type (e.g., forced exercise vs. voluntary exercise) has been found to differentially impact the gut microbiome in mice [163].

In addition to the mode of exercise, which has been found to have varying effects on the gut microbiome in mice if performed voluntarily or forced, it is equally important to consider the optimal dose of exercise in order to maximize the benefits while minimizing potential risks. The specific effects on body composition, cognition, and microbial diversity can vary based on the intensity, duration, frequency, and individual characteristics of the exercise regimen. Further research is needed to better understand the optimal exercise parameters for specific health outcomes, taking into account factors such as inflammation and individual differences. It is worth noting that high-intensity exercise or intensive bouts of unfamiliar exercise, which may lead to excessive production of ROS and GCs, potentially pose risks. This exemplifies Paracelsus’ observation “the dose makes the poison” for exercise. By carefully considering both the mode and dose of exercise, we can gain valuable insights into harnessing the benefits of exercise for cancer survivors. In summary, the amount, duration, and intensity of exercise should be tailored to individual needs, goals, and health status.

The analysis conducted in this paper does not meet the criteria of a systematic review but serves the purpose of providing insights into the underlying mechanisms and proposing avenues for future research. Several significant knowledge gaps persist regarding the pathogenesis of CRF, CRCI, and behavioral changes via the gut–brain axis. While inflammation appears to play a role in all these areas based on existing hypotheses, further validation is needed. Understanding the mechanisms behind commonly experienced symptoms in cancer survivors can guide interventions for more targeted and effective relief. Moreover, additional studies are required to investigate the specific effects of exercise on cachectic cancer patients, secondary sarcopenia due to cancer, and the gut microbiome of cancer survivors. These investigations will help us gain a deeper understanding of how exercise exerts its effects, assess the significance or presence of these effects, and identify opportunities to enhance or replicate them.

The objective of our review was to investigate the mechanisms underlying the beneficial effects of exercise on body composition, cognition and sleep behavior, and gut microbiome health in cancer survivors, with a specific focus on the mechanism of reducing inflammation and oxidative stress. What sets our study apart is its analysis of various exercise types and diverse cancer studies, as presented in Table 1, providing a comprehensive exploration of exercise effects without solely focusing on one type of cancer, one type of exercise, or one specific benefit. The table includes specific human randomized controlled trials and studies that have been examined in this review. However, it is important to note that many other reviews, which mainly focused on specific aspects of the benefits of exercise on cancer survivors, have been used as references throughout our study but are not included in this table [33,37,84,112,154,158]. Through an extensive examination of the mechanisms benefiting healthy individuals or animals, we were able to establish connections to the observed effects of exercise in cancer survivors, offering a comprehensive understanding and valuable insight for this population.

## 4. Conclusions

In conclusion, exercise has emerged as a promising and potent non-pharmacological intervention for cancer survivors, offering multifaceted benefits such as mitigating adverse effects, reducing inflammation and oxidative stress, ultimately improving QOL, and enhancing overall health outcomes in this population. It positively impacts body composition, gut microbiome health, and cognitive function, alleviating fatigue and concentration difficulties. By reducing inflammation, mitigating muscle wasting, and regulating glucocorticoid levels, exercise addresses key factors such as oxidative stress that contribute to disease development and symptoms in cancer survivors. Additionally, exercise promotes the growth of beneficial gut bacteria and decreases the prevalence of harmful bacteria through various mechanisms, including modulating the immune system, reducing inflammation, and improving metabolic function. By enhancing neurogenesis, promoting neuroplasticity through the gut–brain axis, and promoting the release of growth factors, exercise not only reduces the risk of dementia but also alleviates the cognitive impairment commonly experienced by cancer survivors.

Multiple guidelines emphasize the significant health benefits that even a modest amount of physical activity can provide for cancer survivors. It is crucial for cancer survivors to prioritize the incorporation of exercise into their daily routines as early as possible. Additionally, incorporating cognitive training in conjunction with exercise has shown promise in enhancing cognitive function in cancer survivors. To optimize exercise programs for cancer survivors, it is crucial to personalize exercise regimens based on their specific symptoms and requirements. Endurance training exercises such as jogging and cycling have consistently demonstrated their effectiveness in reducing fatigue symptoms, while resistance training has proven reliable in combating cancer cachexia. Healthcare providers should adapt the exercise regimen to align with each patient’s unique symptoms and individual needs, emphasizing exercises that are feasible and offer the most benefits in their particular situation. This tailored approach allows healthcare providers to maximize the benefits and enhance the overall well-being of cancer survivors. Integrating the combination of both endurance and resistance training, as exemplified by the practice of rowing, can serve as a promising starting point in designing exercise programs. However, further research is needed to optimize exercise regimens and gain a deeper understanding of their impact. This knowledge will enable interventions to be more targeted and effective in addressing the specific needs of each patient.

The groundbreaking studies on the replication of exercise-related benefits through targeted administration of BDNF and induction of neurogenesis offer promising avenues for future therapeutic interventions, potentially enhancing cognitive function in cancer survivors and individuals with neurodegenerative diseases. In the future, the replication of other mechanisms explored in this review and their artificial enhancement could further enhance the effects of exercise or be a feasible substitution for exercise, providing additional support and aid for cancer survivors.

## Figures and Tables

**Table 1:** Effects of exercise on cancer survivors: summary of randomized controlled trials and studies examined in this review.

Study	Sample Size	Health Status	Exercise Intervention				Relevant Tests and Measures		Relevant Findings Due to Exercise Intervention	
			Exercise Type	Frequency	Session Time	Total Duration	Measures	Assessments	Beneficial Effects	Unaffected
Brown (2018) [32]	39	Colon cancer (stage I-III)	Moderate:Aerobic, home treadmill	Depends	150 mins OR 300 mins/week	6 months	/	SF-36, PSQI, FSI, FACT-C,	Physical component SF-36, FACT-C, PSQI, and FSI	Mental component SF-36
Courneya (2007) [156]	242	Breast cancer (stage I-IIIa)	Moderate: Resistance OR Aerobic	3x/week	15–45 mins	9–24 weeks	/	FACT-A, self-esteem	Self-esteem	FACT-A
Dieli-Conwrigth (2018) [35]	100	Breast cancer (stage 0-III) BMI > 25.0 kg/m^2^	Moderate to vigorous: Aerobic and resistance	3x/week	150 mins aerobic 2/3 days resistance	16 weeks	**Insulin, leptin, adiponectin:** Blood sample (2x)	/	Reduction in all biomarkers; improved body composition	/
Gavala-González (2021) [54]	28	Breast cancer (stage I-IV)	Moderate:Combined (Rowing)	3x/week	60–90 min	12 weeks	/	IPAQ-SF, SF-36	QOL, overall perceived health, physical activity	/
Himbert (2022) [141]	179	Colon cancer (stage I-IV)	Moderate to vigorous:Various types	Depends	Depends	Depends	**Gut microbiota analysis:** Stool sample (1x)	/	Increased diversity of gut microbiome	/
Ho (2016) [91]	121	Breast cancer	Dance movement therapy	2x/week	90 mins	3 weeks	**Cortisol:** Saliva sample (5x–diurnal slope)	PSQI, BFI	Steeper cortisol slope	/
Hojan (2016) [38]	54	High-risk prostate cancer	Moderate:Aerobic & resistance	5x/week	50 mins	12–20 weeks	**IL-1ß, IL-6, TNF-α:** Blood sample (2x)	FACT-F, QOL (EORTC)	Decreased inflammatory markers; improved fatigue and QOL after RT	Blood markers before RT
Kenfield (2011) [31]	2705	Non-metastatic prostate cancer	General physical activity	Depends	Depends	Depends	**Risk of advanced prostate cancer**	Overall survival	Overall survival; reduced risk of advanced prostate cancer with vigorous activity	/
Lønbro (2013) [69]	41	Head and neck cancer	Progressive resistance training	2–3x/week	Depends	12 weeks	**Body lean mass, muscle strength**	QOL (EORTC)	Lean body mass, muscle strength; QOL cognition	/
Mutrie (2007) [157]	177	Breast cancer (stage 0-III)	Moderate: Walking/cycling/aerobics	3x/week	45 min	12 weeks	**/**	FACT-G, BDI, BMI	Improved BMI and BDI	FACT-G
Oh (2010) [36]	162	Various cancers (mainly breast and colonic cancer)	Medical qigong	2x/week	90 min	10 weeks	**CRP:** Blood sample (2x)	FACT-G, FACT-F, mood state	Reduced CRP; improved QOL and FACT-F, reduced mood disturbance	Anger and hostility, Confusion (mood)
Payne (2008) [43]	20	Breast cancer and hormonal treatment	Moderate: Aerobic, walking activity	4x/week	20 min	14 weeks	**Cortisol, serotonin, bilirubin:** Blood sample (2x)	PSQI, PRFS	Decreased cortisol, serotonin and bilirubin; improved PSQI	/
Ratcliff (2016) [90]	163	Breast cancer (stage 0-III)	Mind–body, yoga	3x/week	60 min	6 weeks	**Cortisol:** Saliva sample (5x– diurnal slope)	PSQI, SF-36	Yoga caused steeper cortisol slope; yoga improved PSQI	PSQI, mental component SF-36
Sampsell (2022) [140]	10	Breast cancer	Mild to moderate: Aerobic and strength	2x/week	60 min	12 weeks	**Gut microbiota analysis:** Stool sample (3x)	FACT-G	Trend toward increased a-diversity between 0 and 12 weeks	FACT-G, human gut microbiome
Smoak (2021) [46]	183	Various cancers	Resistance and aerobic	3x/week	60 min	12 weeks	**BDNF, NGF:** Blood sample (2x)	QOL, fatigue, depression	BDNF increase (in treatment); QOL, fatigue (no treatment)	Depression

Abbreviations: body mass index (BMI), C-reactive protein (CRP), tumor necrosis factor α (TNF-α), Pittsburgh Sleep Quality Index (PSQI), Functional Assessment of Cancer Therapy—General (FACT-G), Functional Assessment of Cancer Therapy—Colorectal (FACT-C), Functional Assessment of Cancer Therapy—Fatigue (FACT-F), Functional Assessment of Cancer Therapy—Anemia (FACT-A), Short Form Survey 36 (SF-36), brief fatigue inventory (BFI), Beck depression inventory (BDI), quality of life (QOL), radio therapy (RT), European organization for research and treatment of cancer (EORTC), brain-derived neurotrophic factor (BDNF), nerve growth factor (NGF), International Physical Activity Short Form (IPAQ-SF).

## Data Availability

Not applicable.
